# Attempted Suicide by Drinking Jeyes' Fluid

**Published:** 1901-07-06

**Authors:** 


### ATTEMPTED SUICIDE BY DRINKING JEYES' FLUID.

Dr. John Woodman[1] records an extraordinary case in which a man with suicidal intent drank a large quantity of Jeyes' Fluid, a quantity estimated at a pint and a quarter. He shortly afterwards became unconscious. The doctor saw him a little less than half an hour after the occurrence, when he lay collapsed, almost pulseless, with stertorous breathing, and with his pupils contracted to a pin's point. A hypodermic injection of brandy was administered, and the stomach tube was passed. On filling the stomach with warm water the doctor was able to pump out a highly concentrated solution of Jeyes' Fluid, and he went on alternately pumping in hot water and pumping out a solution of Jeyes' Fluid for more than two hours, when the water came back almost colourless but with a little blood in it. During this time the man's breathing had become much better and his pulse nearly normal, but he was still unconscious. About seven hours after the stuff had been taken he recovered consciousness. For two days he was only allowed sips of water by the mouth, but after that he was given milk, and at the end of the week he appeared quite well.

[1] Brit. Med. Journ., June 29.

